# Development of numerical processing in children with typical and dyscalculic arithmetic skills—a longitudinal study

**DOI:** 10.3389/fpsyg.2013.00459

**Published:** 2013-07-23

**Authors:** Karin Landerl

**Affiliations:** Department of Psychology, University of GrazGraz, Austria

**Keywords:** dyscalculia, numerical processing development, number comparison, dot counting, number line

## Abstract

Numerical processing has been demonstrated to be closely associated with arithmetic skills, however, our knowledge on the development of the relevant cognitive mechanisms is limited. The present longitudinal study investigated the developmental trajectories of numerical processing in 42 children with age-adequate arithmetic development and 41 children with dyscalculia over a 2-year period from beginning of Grade 2, when children were 7; 6 years old, to beginning of Grade 4. A battery of numerical processing tasks (dot enumeration, non-symbolic and symbolic comparison of one- and two-digit numbers, physical comparison, number line estimation) was given five times during the study (beginning and middle of each school year). Efficiency of numerical processing was a very good indicator of development in numerical processing while within-task effects remained largely constant and showed low long-term stability before middle of Grade 3. Children with dyscalculia showed less efficient numerical processing reflected in specifically prolonged response times. Importantly, they showed consistently larger slopes for dot enumeration in the subitizing range, an untypically large compatibility effect when processing two-digit numbers, and they were consistently less accurate in placing numbers on a number line. Thus, we were able to identify parameters that can be used in future research to characterize numerical processing in typical and dyscalculic development. These parameters can also be helpful for identification of children who struggle in their numerical development.

Efficient processing of numbers and numerical sets in young children has been found to predict later arithmetic skills (Mazzocco and Thompson, 2005; Halberda and Feigenson, 2008; de Smedt et al., 2009; Jordan et al., 2009, 2010; Geary, 2011). There is also converging evidence that numerical processing is deficient in individuals with dyscalculia, a severe and persistent disability in learning arithmetic which can be highly selective, affecting learners with normal intelligence (Butterworth et al., 2011). Basic numerical processing has been proposed to constitute an innate core mechanism which is evident in infants (Xu and Spelke, 2000; Xu and Arriaga, 2007) and underlies all further developments in number processing (Butterworth, 1999; Wilson and Dehaene, 2007; Dehaene, 2011).

Although an association between numerical processing and arithmetic is clearly established, the construct of numerical processing itself is still underspecified: First, various different tasks (shortly described in the next section) have been used to investigate how humans represent and process numbers and numerical sets in their cognitive system. It is as yet unclear which tasks and parameters are best suited to measure typical and atypical developmental trajectories within the domain of numerical processing. Second, up to date, empirical evidence on the development of basic numerical skills comes mostly from cross-sectional studies (Girelli et al., 2000; Holloway and Ansari, 2008; Landerl and Kölle, 2009; Schleifer and Landerl, 2011). In the current study, we repeatedly presented a battery of numerical processing tasks to children with good and poor arithmetic skills during their elementary school years (Grades 2–4), allowing a detailed view on developmental processes. Before explicating the outline of the current study in detail, we will give an overview of the tasks and effects used by previous studies to assess numerical development.

In the dot enumeration paradigm, participants have to count a limited number of dots (usually no more than 10) as quickly as possible. The efficiency of counting procedures increases over time (e.g., Jordan et al., 2006; Reeve et al., 2012). Enumeration tasks induce a characteristic pattern of performance, indicating two distinct enumeration systems (Vetter et al., 2011): small numerosities up to three or four are typically responded to with high accuracy and speed. This process of rapid identification of small dot numbers is termed *subitizing*. When counting higher numerosities, reaction times and error rates rise with increasing numerosity, indicating the execution of a sequential counting procedure. In a recent cross-sectional study, Schleifer and Landerl (2011) found adult-like subitizing performance in 11-year old, but not in younger children. Full competence in sequential counting of larger dot arrays was only evident in 14-year olds, while younger age groups performed at less proficient levels. The only study that assessed dot counting performance longitudinally (7 assessments between the ages of 6 and 11 years) also reported a consistent decrease of response times with increasing age as well as a growing subitizing range (Reeve et al., 2012). While 6-year-old children typically subitized two dots, they were able to subitize three dots by the age of 9. A subitizing range of four dots was not achieved throughout the study. Interestingly, both, Reeve et al. (2012) as well as Schleifer and Landerl (2011) found specific subitizing problems (steeper response time slopes) in poor achievers, while in the counting range, responses were generally slower, but the gradients of response time slopes were similar across achievement groups. These findings suggest that problems in subitizing may be a particularly useful marker of dyscalculia (Butterworth, 1999).

Another simple experimental paradigm that is highly informative with respect to the cognitive representation of number is number comparison. Individuals are asked to select the numerically larger of two numbers or numerical sets (e.g., dot arrays). The speed with which this decision is made depends on the numerical distance between the two numerosities. The smaller this distance, the slower (and less accurately) the decision is made due to a larger internal overlap between the two internal magnitude representations. The acuity of non-symbolic quantity processing increases during development, allowing children to discriminate similar numerical sets more precisely (Halberda and Feigenson, 2008; Piazza et al., 2010). A symbolic distance effect has been demonstrated even among kindergarteners (Sekuler and Mierkiewicz, 1977). Acuity of non-symbolic quantity processing in kindergarten was found to predict arithmetic competence at age six (Mazzocco et al., 2011b) and interindividual differences in the acuity of quantity processing were found to be directly related to arithmetic competence (Libertus et al., 2011). Similarly, Holloway and Ansari (2008) reported a relatively smaller symbolic distance effect in higher grade levels and interpreted this age-related decrease as continuing specification of the cognitive representation of number. In line with this assumption, de Smedt et al. (2009) demonstrated an association between the symbolic distance effect and individual differences in math achievement one year later: Children with a relatively smaller distance effect in grade one had higher math scores in grade two. However, other studies found a rather stable influence of numerical distance on symbolic number comparison across different age or achievement groups, accompanied by a general decrease in response times (Girelli et al., 2000; Landerl and Kölle, 2009; Reeve et al., 2012).

Findings on symbolic and non-symbolic number comparison in dyscalculia are mixed. There is evidence for specific problems in non-symbolic magnitude comparison among dyscalculic individuals (Landerl et al., 2009; Piazza et al., 2010; Kucian et al., 2011; Mazzocco et al., 2011a), however, in some studies the deficits of dyscalculic individuals were limited to symbolic processing of Arabic numbers and did not extend to non-symbolic magnitudes (Rousselle and Noël, 2007; Iuculano et al., 2008; Landerl and Kölle, 2009). Based on this discrepancy, Rousselle and Noël (2007) have suggested that the innate core system of analog magnitude representations in itself may be intact in dyscalculia, but cannot be efficiently accessed from symbolic representations of numbers.

The number comparison paradigm has also been used to investigate the automaticity of numerical processing. When individuals are asked to decide which of two digits is physically larger, numerical value interferes with their physical judgments. Generally, incongruent items (e.g., 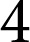 9) are responded to more slowly than congruent items (e.g., 4 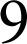; Girelli et al., 2000; Landerl and Kölle, 2009; Bugden and Ansari, 2011). This *size-congruity effect* indicates automatic processing of numbers and requires a certain amount of experience. Cross-sectional studies show interference between physical and numerical size even in first grade (Rubinsten et al., 2002), while in other studies it was not even found in fourth graders (Landerl et al., 2004). Interindividual differences in the degree of automatization and differences in task format make it difficult to compare findings across age groups. A longitudinal design can control for such differences. During development, the size-congruity effect can be expected to become larger as a sign of increasing automatization of numerical processing. Children with dyscalculia are likely to show automatization of numerical processing to a lesser degree or at least at a later developmental stage. Indeed, earlier studies found no (Landerl and Kölle, 2009) or a reduced (Rubinsten and Henik, 2006) size-congruity effect in children with dyscalculia. However, Bugden and Ansari (2011) did not find a correlation of the size-congruity effect with children's arithmetic skills and concluded that automatic processing of numbers is not related to mathematical competence.

Number comparison paradigms with two-digit numbers have been shown to induce a distance effect as well as a compatibility effect (Nuerk et al., 2004). Thus, response accuracy and speed are generally lower when both tens and units are higher in one number (e.g., 83_62, 8 > 6, and 3 > 2) than when tens and units of the two numbers are incompatible (e.g., 82_63, 8 > 6, but 2 < 3). The *compatibility effect* indicates that multi-digit numbers are not processed holistically, but require adequate integration of the composite numerals and their place-value. Acquisition of the place-value system of Arabic numbers is an important step in the development of numerical competences (Mann et al., 2012). Accordingly, first evidence indicates that the compatibility effect is especially marked in young and unexperienced children (Landerl and Kölle, 2009; Pixner et al., 2009) and predicts later arithmetic skills (Moeller et al., 2011). Landerl and Kölle (2009) provided first evidence that the integration of two-digit numbers may pose a particular problem for children with dyscalculia.

A dominant view of the cognitive representation of number is the mental number line model, which postulates that internal representations of numbers and quantities are organized spatially from left to right (Dehaene, 2011). The formation of such a mental number line constitutes a vital step in the development of mathematical skills (Von Aster and Shalev, 2007). In order to investigate the format of these mental representations, children are asked to place particular numbers on lines with endpoints of 0 and 100, respectively, 0 and 1000. A standard finding is that young children overestimate the numerical size of small numbers, inducing a logarithmic number line function. With increasing experience, children's estimates become more realistic, shifting the function from a logarithmic to a linear curve (Siegler and Opfer, 2003; Siegler and Booth, 2004; Booth and Siegler, 2006; Berteletti et al., 2010; but see Ebersbach et al., 2008; Moeller et al., 2009; Barth and Paladino, 2011 for different interpretations). Early competence in the number line task predicts later arithmetic skills (Geary, 2011), and benefits from number line trainings on children's mental representation of number and their arithmetic competence have been demonstrated (Siegler and Ramani, 2009; Kucian et al., 2011). Children with dyscalculia have been found to be less precise in their estimations and there is some indication that this is partly due to a delay in the logarithmic-linear shift (Landerl et al., 2009).

To sum up, a variation of different paradigms has been used to investigate the development of the cognitive representation of number. The general pattern is that the representational system of numbers and numerosities becomes more precise and more efficient during typical development, while this is not the case (at least not to the same extent) in dyscalculic individuals. However, integrating findings from different studies is often problematic due to variations in methodology and sample selection criteria. As most evidence on the development of numerical competencies comes from cross-sectional studies, current knowledge on the stability of task performance across time is scarce. Only Reeve et al. (2012) report reasonable stability of dot enumeration and symbolic number comparison. In latent cluster analyses, children were categorized into slow, medium, and fast subgroups based on their task-specific response times. Over seven assessments carried out between the ages of 6 and 11 years, 69% of a random sample of 159 children remained in the same cluster subgroup and no child changed from the medium or fast groups to the slow group. Still, this finding implies that almost one third of the sample did change subgroup at least once. Ordinal correlations of task-specific group membership at the different assessment points were significant (with the exception of number comparison at the age of 6, which may have overstrained some children), but mostly below 0.7 before the age of nine and a half.

The current study also aimed at investigating developmental trajectories of numerical processing and their stability over a longer time period. In order to get a broader picture of numerical development, we decided to use a range of standard numerical tasks that are assumed to tap into different aspects of numerical cognition. As we were particularly interested in differences in numerical development between children with typical arithmetic development and children with dyscalculia, we started our study at the end of grade 1 and selected participants based on their maths performance after 1 year of formal teaching and during the following 2 years. Children with typical arithmetic development and children with marked and persistent problems in this domain were followed longitudinally over a 2-year period and performed the numerical task battery five times throughout the study.

In particular, our research questions were: (1) How do standard effects of numerical processing develop in children with age-adequate arithmetic skills? (2) Are children with dyscalculia different from typically developing children in all numerical processing tasks, or is there a dyscalculia-specific profile? (3) Is the developmental trajectory of dyscalculia mostly delayed or are there characteristic deficiencies that cannot be explained by a general slowness in the acquisition of arithmetic skills? (4) How stable is the development of numerical processing during the elementary school years?

## Materials and methods

### Design

The present longitudinal study investigated the development of childrens' numerical skills from the beginning of Grade 2 to the beginning of Grade 4. Based on a screening at the end of Grade 1 and their performance during the study period, children were allocated to a group with age-adequate and a group with atypically poor arithmetic development. Two times per school year (October and March), children were individually tested with a computerized battery of numerical tasks, resulting in five assessment points altogether (t1–t5). Results from the screening period at the end of Grade 1 and the first individual assessment two months later at the beginning of Grade 2 are reported as one assessment point (t1).

### Participants

The 83 participants of the current analysis (42 children with age-adequate arithmetic development and 41 children with dyscalculia) were selected as follows: during a screening period, a classroom test of arithmetic (Haffner et al., 2005) was given to 505 children at the end of first grade attending 19 different elementary schools in a south-western area of Germany. All children who performed more than 1 *SD* below age expectations on this test were invited for further assessment. For each child with poor arithmetic skills, another child was randomly selected from the same classroom who did not show any particular arithmetic problems (test performance not more than 0.5 *SD*s below the age norm). In order to rule out more general learning problems, the following exclusion criteria were applied:

– performance more than 1 *SD* below the age norm on a standardized test of nonverbal IQ (CFT 1; Cattell et al., 1997).
– performance more than 1 *SD* below age norm on a test of verbal short-term memory (German version of WISC IV digit span forward and backwards, Petermann and Petermann, 2008).
– clinical diagnosis of ADHD or performance more than 1.5 *SD*s below age norm on a standardized attention test (KITAP, Zimmermann et al., 2002) in order to avoid as far as possible confounds from comorbid attentional deficits.
– performance more than 2 *SD*s below the age norm on a standardized test of word reading (SLS 1–4, Mayringer and Wimmer, 2003) administered at the beginning of Grade 2 in order to avoid as far as possible confounds from comorbid dyslexia. Current evidence (e.g., Landerl et al., 2009) suggests that the cognitive deficits typically associated with dyslexia are largely independent from numerical processing, therefore, we decided to apply a rather conservative exclusion criterion for reading deficits.

Altogether, 139 children were followed over the whole study period. During the study period, the standardized test of arithmetic (HRT 1–4, see below) was given three times, i.e., end of Grade 1 and beginning of Grades 3 and 4. The two groups reported here were selected from the full longitudinal sample based on the following criteria: Children with age-adequate arithmetic development had to show at least average performance (not more than 0.5 *SD* below age norms) in all three arithmetic assessments. Children with dyscalculia showed persistent problems in arithmetic during the whole study period. At t1, all children of this group performed more than 1 *SD* below the age norm. At the latter two assessment points, performance was never better than 0.5 *SD* below the age norm. Table 1 shows that at all three assessment points, the average performance of the dyscalculia group was markedly deficient with about 1.5 *SD*s below age norm^1^.

**Table 1:** **Participants' details**.

	**Typically developing (*n* = 42) Mean (*SD*)**	**Dyscalculic (*n* = 41) Mean (*SD*)**	***d***
Percentage boys	57.10	39.00	
Age (months)	91.42 (3.80)	90.07 (4.75)	
Nonverbal IQ	115.57 (11.91)	101.93 (9.45)	1.08^**^
Verbal working memory^a^	11.02 (2.27)	9.83 (1.95)	0.54^*^
Attention^b^	49.24 (4.29)	45.19 (3.42)	0.93^**^
Reading^c^	109,69 (14.02)	90.37 (14.89)	1.11^**^
**ARITHMETIC^b^**			
T1	55.21 (7.76)	34.27 (5.32)	3.15^**^
T3	57.4 (6.5)	37.32 (4.95)	3.48^**^
T5	56.17 (6.22)	36.9 (4.72)	3.49^**^
**ADDITION^d^**			
T1	17.6 (3.39)	8.83 (3.03)	2.73^**^
T3	24.74 (3.8)	16.15 (2.74)	2.60^**^
T5	29.12 (3.05)	20.63 (3.48)	2.60^**^
**SUBTRACTION^d^**			
T1	16.48 (4.31)	7.54 (3.58)	2.26^**^
T3	24.67 (4.62)	13.61 (4.32)	2.07^**^
T5	28.83 (3.35)	18.41 (4.59)	2.65^**^
**MULTIPLICATION^d^**			
T3	20.29 (4.5)	11.24 (4.73)	1.96^**^
T5	25.17 (4.56)	15.32 (3.98)	2.30^**^
**DIVISION^d^**			
T3	18.07 (5.26)	6.98 (4.84)	2.20^**^
T5	24.93 (4.95)	12.32 (5.68)	2.37^**^

a Standard Score (M: 10 / SD: 3).

b t-Score (M: 50 / SD: 10).

c Reading Quotient (M: 100 / SD: 15).

d Raw score (number of correct responses given in 2 min).

* p < 0.05;

** p < 0.01.

### Tasks

#### Standardized tests

***Arithmetic.*** A standardized test of arithmetic skills, the subtests of the subscale “arithmetic operations” of the Heidelberger Rechentest (HRT 1–4) (Haffner et al., 2005), was given three times during the project period (t1: end of Grade 1, t3: beginning of Grade 3 and t5: beginning of Grade 4). At t1, children's competence in mental calculation (addition and subtraction) was assessed by specific subtests requiring children to write down as many correct answers as possible to a list of calculations (gradually increasing in difficulty) within a time limit of 2 min. Two further subtests had a slightly more complex format, but with the same 2 min time restriction (e.g., “__ −2 = 6” – supply: “4”; “9 + 1 __ 11” – supply “<”). At the later assessment points, multiplication and division were also assessed by 2 min subtests. The dependent measure was the number of correct responses combined for all subtests.

***Nonverbal IQ.*** The CFT1 (Cattell et al., 1997) was given at the end of first Grade (t1). This test is based on Cattell's Culture Free Intelligence Test, Scale 1 (Cattell, 1950) and consists of five subtests (Substitutions, Mazes, Classifications, Similarities, and Matrices).

***Working memory.*** The subtest *digit span* (forward and backwards) of the German version of the Wechsler Intelligence Scale for Children (Petermann and Petermann, 2008) was given during the screening period at the end of Grade 1.

***Attention.*** A standardized computer-test battery assessing different aspects of attention (Zimmermann et al., 2002) was carried out in the middle of Grade 2 (t3). Five subtests measured children's alertness, attentional flexibility, distractibility, sustained attention, and divided attention.

***Reading.*** At t1, a standardized reading test (Mayringer and Wimmer, 2003) was given in which children had to silently read simple sentences and mark whether the content of the sentence was right or wrong. The main criterion is reading speed, more specifically the number of correctly marked sentences within a time limit of 3 min.

#### Numerical processing

All numerical processing tasks were presented on notebooks running Presentation software. In all tasks, the background of the screen was black and the items were presented in white color in the middle of the screen. Participants were tested individually in a quiet room in school.

***Dot enumeration.*** Sets of randomly arranged dots ranging from one to eight were presented which children had to enumerate as quickly as possible. The response was given by simultaneously pressing the space button and pronouncing the number. The experimenter recorded correctness. The key press initiated a mask (block pattern) for 1500 ms which prevented counting based on an after image of the dot display. The 48 trials (six per dot number) were presented in a fixed pseudo-random order with the proviso that no dot number occurred twice in succession (interstimulus-interval: 1120 ms).

***Single digit comparison.*** Children were presented with 56 pairs of digits and selected as quickly as possible the numerically larger one by pressing the corresponding keyboard button. Numerical distances ranged from 1 to 8 (16 trials for distance 1, ten trials for distance 2–3, and four trials each for distances 4–8). Stimuli were written in a 36-point Times New Roman font and presented in a randomized order, beginning with six practice trials (interstimulus-interval: 560 ms).

**Figure 1:**
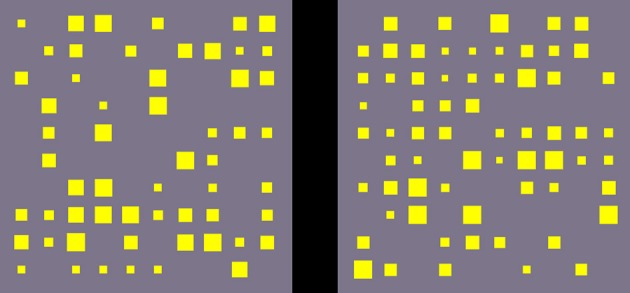
**Example item of the magnitude comparison task (small distance)**.

***Magnitude comparison.*** Two gray displays with different numbers of yellow squares appeared side by side on the screen and children selected the numerically larger one as quickly as possible by keypress response (see Figure 1). Displays presented between 20 and 72 squares, and numerical distances between the two displays ranged from eight to 25 squares. Relatively high numerosities ensured that children based their decisions on estimation and not on verbal counting. The total surface areas in the two displays were identical. Each display consisted of different square sizes to avoid displays with larger numerosities systematically consisting of smaller squares. After three practice trials with feedback, 72 test trials (four for each numerical distance) were presented in a fixed pseudo-random order (interstimulus-interval: 300 ms).

***Physical comparison.*** Here, children had to select the physically larger of two Arabic digits while ignoring their numerical value. In 32 trials, physical and numerical size were congruent (e.g., 2 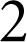), in further 32 items physical and numerical size were incongruent (e.g., 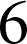 6), and 18 neutral items displayed the same digit twice in different sizes (e.g., 2 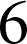). Print sizes were a 48- and 24-point font. After six practice trials, the items were presented in random order (interstimulus-interval: 560 ms).

***Comparison of two-digit numbers.*** Children were asked to select the numerically larger of two two-digit numbers between 21 and 98. In 30 items, both decade and unit digit were larger in one number (compatible items, e.g., 41 75), in further 30 items, the decade digit was larger in one and the unit digit was larger in the other number (incompatible items, e.g., 41 26). Overall distance and problem size were matched between the two compatibility conditions. All items had small numerical distance between the decade digits and large distance between the unit digits (e.g., 37 52) as previous evidence (Nuerk et al., 2004) suggested that a compatibility effect was most likely to appear under these conditions. Twenty neutral items only differing in the unit digit (e.g., 61 68) were included in order to prevent children from basing their decisions on the decade digits only. Items were presented in a random sequence (interstimulus-interval: 560 ms).

***Number line task.*** This task was adapted from Siegler and Opfer's (2003) number-to-position task. A number line (25 cm) was presented with the left end always labeled “0,”and the right end labeled “100” for the first 24 items and “1000” for the next 24 items. Numbers in the lower range were overrepresented to allow discrimination between logarithmic and linear functions. An Arabic number appeared on top of the screen, and children read it out loud. Transcoding errors (which were exceptional) were corrected by the experimenter. Children indicated where the number would fall on the line by pointing with a cotton bud. The experimenter placed the cursor on this position and clicked the mouse. The deviance from the precise position was calculated in pixels (1 cm corresponded to 37.5 pixels). Each condition was introduced by three practice items. At t1 (beginning of Grade 2), only the number line 0–100 was given as the number line 0–1000 was considered too difficult.

## Results

### Arithmetic performance

Table 1 presents for each of the three assessment points (end of Grade 1, beginning of Grades 3 and 4) the overall test scores for the two groups which constituted the selection criterion. Table 1 also presents the raw scores for four of the arithmetic subtests, representing the number of simple calculation problems were answered correctly within two minutes. These subtest raw scores indicate a dramatic difference between the two groups: Dyscalculic children's scores were consistently more than 2 *SDs* below the number of items that were solved by children with age-adequate development.

### Nonverbal intelligence, verbal working memory, attention, and reading

Since low performance in any of these standardized tests was used as an exclusion criterion, performance of all participants was within average range. Still, average performance of the typically developing group was significantly better compared to the dyscalculia group on each of these measures.

### Numerical processing

Reliability was very high for all response time based measures (Cronbach's Alpha ranging between 0.93 and 0.98 for RTs at each assessment point) and sufficiently high for the two mental number line conditions (between 0.72 and 0.90, all *p*s < 0.01). Individual median reaction times were calculated for each child in each condition. Only reaction times for correct responses were considered, and reaction times lower than 200 ms and higher than 10000 ms were excluded. With the exception of the number line task where mean deviance scores were analysed, the main dependent variable used in statistical analysis was inverse efficiency (IE), which combines accuracy and speed of response into one measure by dividing the adjusted median reaction times by the proportion of correct responses (Bruyer and Brysbaert, 2011).

Statistical analysis of each task was achieved by ANOVAs including all relevant within-task factors as well as assessment point (t1–t5) as within-subjects factors and arithmetic level (typical vs. dyscalculic) as between-subjects factor. In case of violation of sphericity, Greenhouse-Geisser correction was applied. Significant effects were followed up by paired comparisons under Bonferroni correction. Stability of task performance across the study period was examined by inspecting the correlational patterns between the five assessment points.

### Efficiency of numerical processing

**Figure 2:**
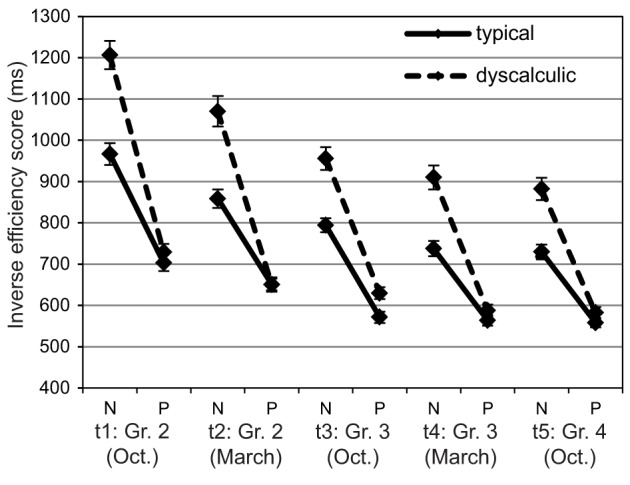
**Efficiency of numerical processing: Mean IE-scores (SEs as error bars) for numerical (N) and physical (P) comparison across five assessment points**.

First, we wanted to know whether the efficiency to process numbers developed specifically or whether it was mainly dependent on increases in general processing speed. Figure 2 presents for each assessment point children's IE-scores in the neutral condition of the physical comparison task with the average IE-scores in the digit comparison task. Although numbers are presented in the physical comparison task, the neutral condition requires a decision based on the physical size of two identical digits (e.g., 2 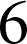) and therefore provides a non-numerical control measure of children's efficiency to perform forced-choice paradigms. Response accuracy was close to ceiling in both conditions even at t1 so that IE-scores mostly represented response times. Children showed systematically higher IE-scores in the numerical condition than in the physical condition, *F*_(1, 80)_ = 458.65, *p* = <0.01; η^2^ = 0.85, IES-scores decreased systematically over time, *F*_(2.81, 224.57)_ = 161.18, *p* < 0.01; η^2^ = 0.67 (all *p*s < 0.05) and dyscalculic children showed generally higher IE-scores than typically developing children, *F*_(1, 80)_ = 23.29, *p* < 0.01; η^2^ = 0.23. Figure 2 shows that IE-scores of dyscalculic and typically developing children were similar in the non-numerical condition (all *p*s > 0.05). In the numerical condition, however, dyscalculic children showed clearly higher IE-scores than their typically developing peers, resulting in a significant task × arithmetic level interaction, *F*_(1, 80)_ = 35.78, *p* < 0.01, η^2^ = 0.31. The interactions task × assessment point and task × assessment × arithmetic level were also reliable, *F*_(2.94, 244.57)_ = 24.28, *p* < 0.01; η^2^ = 0.23, and *F*_(2.94, 234.85)_ = 0.4.95, *p* < 0.05.; η^2^ = 0.06. *Post-hoc* analysis indicated that the difference in IE-scores between the two conditions decreased systematically over time among dyscalculic children (all *p*s < 0.001 except t1 vs. t2 and t4 vs. t5 where *p* = 0.07 and t3 vs. t4 and t5 where *p* > 0.1). The developmental change of these difference scores was smaller and not always significant among the typically developing children (*p* < 0.05 for t1 > t2, t4, t5, and t3 > t4, t5).

The stability of the efficiency of numerical processing across the study period was confirmed by mostly moderate correlations (ranging between 0.53 and 0.79, *p* < 0.001) among the difference scores (numerical minus physical condition) at each assessment point. The correlation between t1 and t4 appeared to be somewhat lower (0.23, *p* = 0.03) and the correlation between the two final assessment points (t4 and t5) was particularly high (0.84).

### Dot enumeration

**Figure 3:**
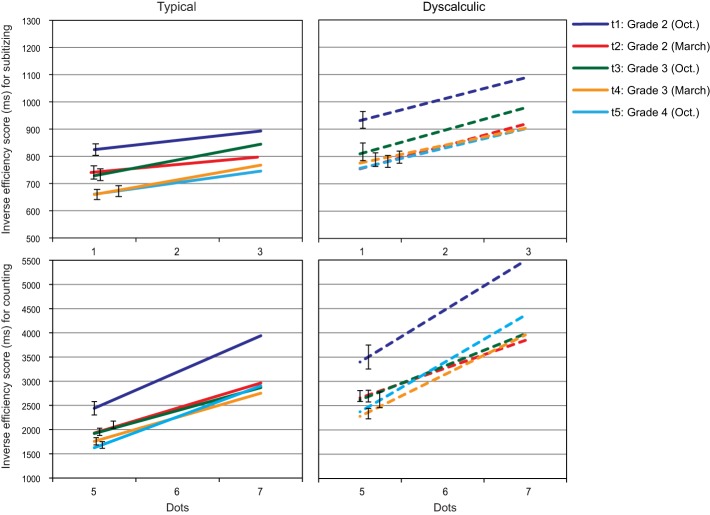
**Dot enumeration: Regression lines for IE-scores in the subitizing range (top) and the counting range (bottom) across five assessment points (error bars represent SEs of intercepts)**.

For each child, the best fitting regression lines were calculated separately for the subitizing range (1–3) and the counting range (5–7)^2^. The regression lines are presented in Figure 3.

Because of very obvious and expected differences between IE-scores for subitizing and counting, intercepts and slopes for these two numerical ranges were analysed separately. In the subitizing range, the main effect of assessment point was significant for intercepts, *F*_(3.07, 242.24)_ = 25.40, *p* < 0.01; η^2^ = 0.243, while slopes remained largely constant across the study period, *F*_(3.07, 242.11)_ = 0.52, n.s.; η^2^ = 0.007. Interestingly, intercepts as well as slopes turned out to be larger in the dyscalculic than in the typically developing group, intercepts: *F*_(1, 79)_ = 10.55, *p* = 0.002; η^2^ = 0.12, slopes: *F*_(1, 79)_ = 10.36, *p* < 0.01; η^2^ = 0.12. The interaction approached significance for intercepts, *F*_(3.07, 242.24)_ = 2.40, *p* = 0.067; η^2^ = 0.030, but not for slopes, *F*_(3.06, 242.11)_ = 0.98, n.s.; η^2^ = 0.012. *Post-hoc* analysis indicated larger subitizing intercepts at t1 than at all later assessment points for the dyscalculia group while the decrease in intercepts over time was more systematic in the typically developing group (t1 > t2, t4, t5; t2 > t4, t5, t3 > t5).

In the counting range, the main effect of assessment point was again significant for intercepts, *F*_(2.27, 179.33)_ = 23.31, *p* < 0.05.; η^2^ = 0.228 (*post-hoc* tests: t1 < t2, t3 < t4, t5, all *p*s < 0.06), but not for slopes, *F*_(1.68, 132.22)_ = 2.11, n.s.; η^2^ = 0.026. Intercepts were significantly larger in the dyscalculia than in the typically developing group, *F*_(1, 79)_ = 34.05, *p* < 0.05.; η^2^ = 0.301. The effect of arithmetic level was only of borderline significance for the counting slopes: *F*_(1, 79)_ = 3.86, *p* = 0.053; η^2^ = 0.047. No assessment point × arithmetic level interactions were evident for the counting range (*F*s = 1.12 and 0.33, n.s.).

Low to medium range correlations were observed among the intercepts of the first four assessment points for both, subitizing (0.28, *p* = 0.011 to 0.67, *p* < 0.001) and counting (0.32, *p* = 0.003 to 0.64, *p* < 0.001). Stability was generally low for subitizing slopes across the first four assessment points, with correlations in the moderate range (0.22–0.34, *p*s < 0.05, except t1 and t3 with *r* = 0.15, n.s.). Counting slopes showed some stability between t1 and t2 (0.28, *p* = 0.011), while no significant correlations were evident between t2, t3, and t4. Higher stability for the dot counting task was found between t4 and t5, with high correlations between the intercepts for subitizing (0.78) and counting (0.80), as well as between the counting slopes (0.90, all *p*s < 0.001). The correlation between the subitizing slopes at t4 and t5 was in the medium range with *r* = 0.38, *p* < 0.001.

### Single digit comparison^3^

**Figure 4:**
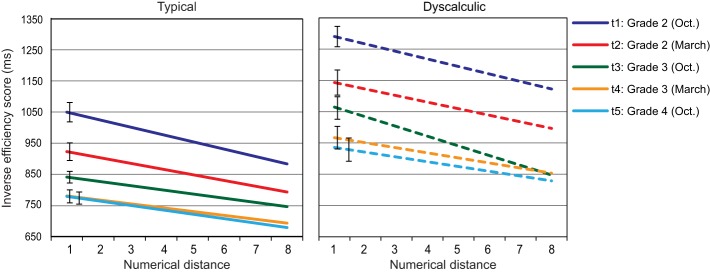
**Single digit comparison: regression lines of IE-scores in relation to numerical distance across five assessment points (error bars represent SEs of intercepts)**.

In order to display the effect of numerical distance, the best fitting regression line was calculated for each child. For intercepts, both main effects were significant, assessment point: *F*_(2.94, 235.41)_ = 87.47, *p* < 0.01; η^2^ = 0.55; arithmetic level: *F*_(1, 80)_ = 37.00, *p* < 0.01; η^2^ = 0.82. Figure 4 shows a continuous decrease of intercepts from t1 to t5 (t1, t2 > t3, t4, and t3 > t5; *p*s < 0.05). Most importantly, intercepts of the dyscalculia group were consistently higher than those of the control group at each assessment point (all *p*s < 0.01).

For slopes, the main effect of assessment point was significant, *F*_(2.96, 236.51)_ = 3.48, *p* < 0.05; η^2^ = 0.042, and the interaction assessment point × arithmetic level approached significance: *F*_(2.96, 236.51)_ = 2.35, *p* = 0.074; η^2^ = 0.029. Visual inspection of Figure 4 as well as *Post-hoc* analysis indicated that this interaction was driven by a group difference at t3 (*p* < 0.001), but not at the other assessment points (all *p*s > 0.05).

Thus, although the overall efficiency of numerical processing (represented by intercepts) showed a clear improvement across the study period and a considerable developmental delay for the dyscalculia group, we could not confirm earlier evidence from cross-sectional studies (Holloway and Ansari, 2008; Landerl and Kölle, 2009) reporting a decrease in numerical distance effect over time. One could even argue that there was a relative increase of the distance effect when taking the decrease in intercepts into consideration. However, when this was examined in an additional ANOVA where slopes divided by intercepts were subjected as dependent measures, no significant effects remained.

Robust correlations ranging between 0.62 and 0.90 (all *p*s < 0.001) were observed between the intercepts of the five assessment points. For slopes, however, a reasonable amount of stability was only evident from t3 on (0.36, 0.52, and 0.64 for correlations between t3 and t4, t3 and t5 and t4 and t5, respectively, all *p*s < 0.001), while no significant correlations were found with the earlier assessment points.

### Magnitude comparison^4^

**Figure 5:**
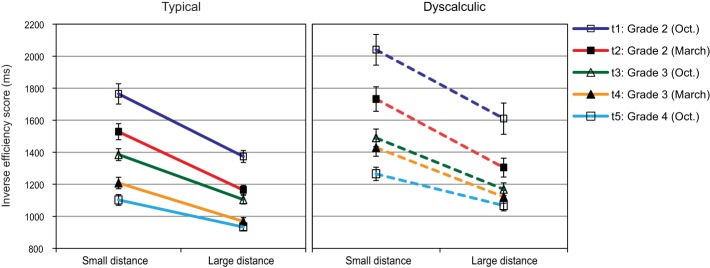
**Magnitude comparison: Mean IE-scores (SEs as error bars) for small and large numerical distance items at each assessment point**.

In order to investigate the non-symbolic distance effect the items of this task were grouped into two distance levels: (1) small distance condition (differences between the two displays ranged from 8 to 16) and (2) large distance condition (differences between the two displays ranged from 17 to 25). From Figure 5, it is obvious that all groups were faster in responding to large distance items than to small distance items. In a 2 (distance) × 5 (assessment point) × 2 (arithmetic level) ANOVA, the main effect of distance, *F*_(1, 81)_= 477.04, *p* < 0.01; η^2^ = 0.86, was indeed highly reliable. The main effects of assessment point, *F*_(2.22, 179.68)_ = 81.97, *p* < 0.01; η^2^ = 0.50 was modified by a significant interaction distance × assessment point, *F*_(3.01, 243.82)_ = 19.58, *p* < 0.01; η^2^ = 0.20. *Post-hoc* analysis indicated significant differences between all assessment points for small as well as large distance items (*p*s < 0.01). The interaction was caused by a relatively small decrease in IE-scores from t4 to t5 for items with a large numerical distance.

Importantly, there was an effect of arithmetic level, *F*_(1, 81)_ = 11.17, *p* = 0.03; η^2^ = 0.12, which did not interact with the other factors, indicating that the dyscalculia group showed lower performance over all, while the pattern of performance was comparable throughout the study period. This was confirmed in a final ANOVA calculating a relative distance effect as percent increase of IE-scores in the small compared to the large distance condition. In this analysis, only the main effect of assessment point remained significant, *F*_(3.31, 268.10)_ = 8.63, *p* < 0.01; η^2^ = 0.10.

There were medium-sized correlations among IE-scores for small as well as large distance items between t1 and t4 (ranging from 0.38 to 0.56, all *p*s < 0.001) and high correlations between t4 and t5 (0.95 for both, small and large distance items). For the non-symbolic distance effect itself, only moderate correlations were observed between t1 and the later assessment points (*r*s between 0.20, *p* = 0.07, and 0.38, *p* < 0.001) and t3 and the later assessment points (*r* = 0.21, *p* = 0.06 for t4 and 0.30, *p* = 0.007 for t5). High stability for the non-symbolic distance effect was only achieved between the final two assessment points (*r* = 0.76, *p* < 0.001).

### Physical comparison

**Figure 6:**
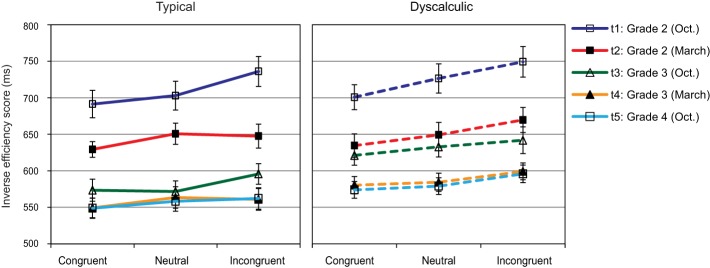
**Physical comparison: Mean IE-scores (SEs as error bars) for congruent, neutral, and incongruent items at each assessment point**.

Figure 6 shows a very systematic size congruity effect for both typically developing and dyscalculic children. In a 3 (congruity) × 5 (assessment point) × 2 (arithmetic level) ANOVA we found significant main effects of congruity, *F*_(1.96, 158.43)_ = 34.91, *p* < 0.01; η^2^ = 0.30, and assessment point, *F*_(2.77, 224.53)_ = 79.11, *p* < 0.01; η^2^ = 0.48, but no difference between groups and no interactions involving group. *Post-hoc* analyses showed significantly lower IE-scores for congruent than for neutral items (facilitation effect), and again lower IE-scores for neutral than for incongruent items (interference effect) (*p*s < 0.01). IE-scores decreased systematically from t1 to t5 (all *p*s < 0.05). The only significant interaction was found between congruity and assessment point: *F*_(5.61, 454.45)_ = 2.23, *p* = 0.043; η^2^ = 0.03. Pairwise contrasts indicated a significant difference between t3 and t4 in the congruent and the incongruent, but not in the neutral condition.

Thus, the interesting finding from this task was that even at the earliest assessment point at the beginning of Grade 2, typically developing and even dyscalculic children showed a significant influence of the numerical value of two presented digits on their non-numerical decision. In contrast to our expectation that the size of the facilitation and interference effects should increase with experience, these effects did not change much across the whole period of the study, in spite of a general incease in processing efficiency. In a final analysis, it was examined whether there were relative differences in the facilitaion and interference effect when these effects were expressed as % change of IE-scores in relation to the neutral condition. Mostly because variability between participants was high, none of the effects remained significant.

Although there were moderate correlations among the first four assessment points for IES-scores in each of the three conditions (*r*s between 0.36 and 0.55, all *p*s < 0.001) and high correlations between t4 and t5 (congruent: 0.96, neutral: 0.95, incongruent: 0.96), the only correlations over time for the facilitation and the interference effect became evident between t4 and t5 (0.71 and 0.73, *p* < 0.001).

### Comparison of two-digit numbers

**Figure 7:**
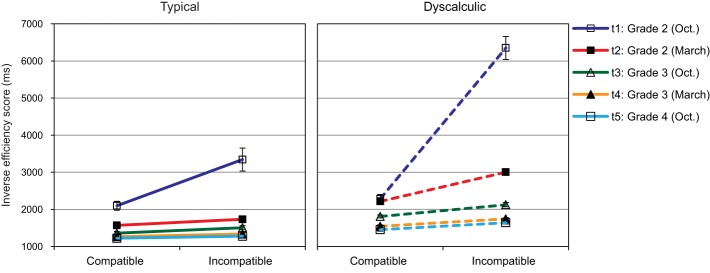
**Comparison of two digit numbers: Mean IE-scores (SEs as error bars) for compatible and incompatible items across five assessment points**.

This was the only task in our numerical processing battery for which response accuracy was not close to ceiling and therefore had a considerable impact on IE-scores. Especially at t1, both groups showed considerable problems with the incompatible condition with 73.73% correct for typically developing and only 50.57% correct for the dyscalculia group. Typically developing children showed mean response accuracies above 90% for later assessments of incompatible items and for compatible items throughout. The dyscalculia group reached this high level of performance only at t4 for incompatible items (88.20% correct) and even in the compatible condition, only 87.37% of the items were responded to correctly at t1. IE-scores, which integrate these accuracy scores with children's speed of response, are plotted in Figure 7.

In a 2 (compatibility) × 5 (assessment point) × 2 (arithmetic level) ANOVA, all main effects were significant: compatibility, *F*_(1, 81)_ = 56.42, *p* < 0.01; η^2^ = 0.41, assessment point, *F*_(1.19, 96.20)_ = 59.15, *p* < 0.01; η^2^ = 0.42, arithmetic level, *F*_(1, 81)_ = 32.08, *p* < 0.01; η^2^ = 0.28. Overall, children had higher IE-scores in the incompatible than in the compatible condition. IE-scores decreased systematically during the study period (t1 > t2, t3 > t4 > t5, all *p*s < 0.01), and children with dyscalculia had higher IE-scores than typically developing children. In addition, all interactions were reliable, compatibility × assessment point: *F*_(1.15, 92.76)_ = 24.19, *p* < 0.01; η^2^ = 0.23, compatibility × arithmetic level: *F*_(1, 81)_ = 16.17, *p* < 0.01; η^2^ = 0.17, assessment point × arithmetic level: *F*_(1.19, 96.20)_ = 5.70, *p* < 0.05; η^2^ = 0.07, compatibility × assessment point × arithmetic level: *F*_(1.145, 92.76)_ = 6.88, *p* < 0.01; η^2^ = 0.78.

In order to interpret this complex pattern of interactions and to analyze the particular problems children face when processing two-digit numbers, two additional ANOVAs were calculated: First, we subjected the IE-scores for the easier condition of compatible items to a 5 (assessment point) × 2 (arithmetic level) ANOVA. Both main effects were reliable, assessment point: *F*_(2.18, 176.20)_ = 61.38, *p* < 0.01; η^2^ = 0.43, arithmetic level: *F*_(1, 81)_ = 21.68, *p* < 0.01; η^2^ = 0.21, and the two factors also interacted, *F*_(2.18, 176.20)_ = 4.23, *p* < 0.05; η^2^ = 0.05. *Post-hoc* analysis indicated a systematic decrease of IE-scores during the study period for the dyscalculic children (all *p*s = 0.001 except t1 vs. t2), while among typically developing children the IE-score differences between adjacent assessment points were too small to be reliable from t2 on (all *p*s < 0.06 except t2 vs. t3, t3 vs. t4 and t4 vs. t5). The difference between the two groups was significant at all assessment points except t1, which is obviously due to the high variability (especially among the dyscalculic children).

The developmental trajectories of the compatibility effect were analysed by subtracting IE-scores for compatible items from those for incompatible items. This difference score was again subjected to a 5 (assessment point) × 2 (arithmetic level) ANOVA. Again, both main effects and the interaction were reliable, assessment point: *F*_(1.15, 92.76)_ = 24.19, *p* < 0.01; η^2^ = 0.21, arithmetic level: *F*_(1, 81)_ = 16.17, *p* < 0.01; η^2^ = 0.17, assessment point × arithmetic level: *F*_(1.15, 92.76)_ = 6.88, *p* < 0.01; η^2^ = 0.08. As evident from Figure 7, both groups showed an especially strong compatibility effect at t1, but this effect was still significantly larger (*p* < 0.01) for dyscalculic (4067) than for typically developing children (1246 ms). Among the typically developing children the compatibility effect was relatively small and similar for the later assessment points (t2: 163 ms; t3: 144 ms: t4: 71 ms, t5: 55 ms). In the dyscalculia group, however, particular problems to integrate the tens and units of two-digit numbers were still evident at t2: Their compatibility effect (789 ms) was significantly larger compared to typically developing children at t2 and larger than at the later assessment points (t3: 316 ms, *p* = 0.07; t4: 200 ms, *p* = 0.019; t5: 185 ms, *p* = 0.013). Group differences between typically developing and dyscalculic children were still marked at the later assessment points, t3: *p* = 0.08; t4 and t5: *p* < 0.05.

In summary, these longitudinal data clearly showed that efficient processing of two-digit numbers develops slowly and poses a particular challenge to children with dyscalculia. Correlations of IE-scores for incompatible items were only moderate for t1 with later assessment points (between 0.32 and 0.34, *p* = 0.002) and in the medium range for t2, t3, and t4 as well as for compatible items among the first four assessment points (*r*s between 0.48 and 0.77). Correlations were considerably higher between t4 and t5 (0.95 and 0.91 for compatible and incompatible items, respectively). For the compatibility effect itself, moderate correlations were found between t2 and t3 (*r* = 0.37, *p* = 0.001), t3 and t4 (*r* = 0.24, *p* = 0.026). Once again, reasonable stability was only evident between t4 and t5, *r* = 0.73, *p* < 0.001.

### Number line task

**Figure 8:**
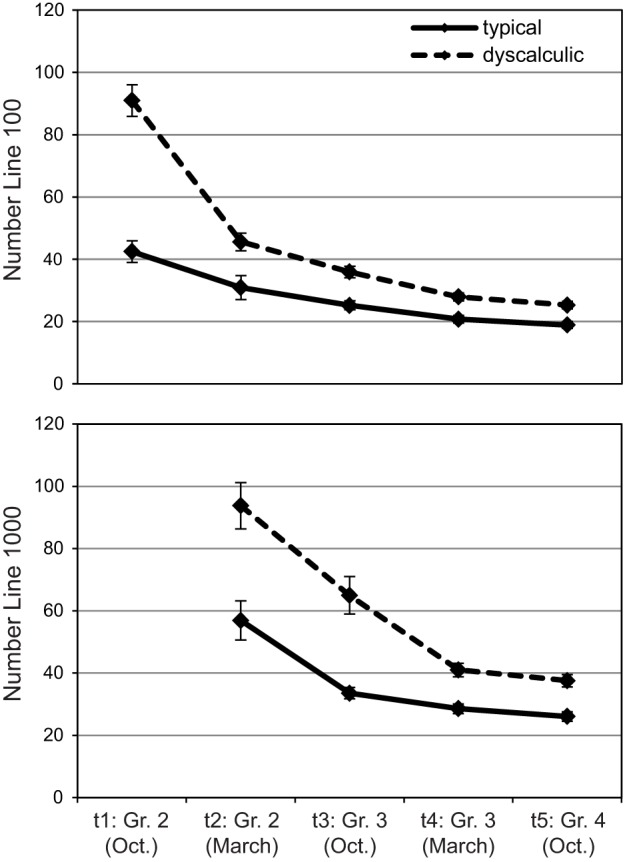
**Number line: Mean deviance scores in pixel (SEs as error bars) across five assessment points for number lines 100 (top) and 1000 (bottom)**.

Two separate ANOVAs with median deviance in pixel (see Figure 8) as dependent variable were calculated for number lines 0–100 and 0–1000. For number line 0–100, assessment point as well as arithmetic level showed reliable effects, *F*_(2.18, 176.16)_ = 121.93, *p* < 0.01; η^2^ = 0.60, and *F*_(1, 81)_ = 53.37, *p* < 0.01; η^2^ = 0.38, which were modulated by a significant interaction, *F*_(2.18, 176.16)_ = 29.03, *p* < 0.01; η^2^ = 0.26. Children with dyscalculia showed higher deviance scores than their typically developing peers at all assessment points (all *p*s < 0.05), but this group difference decreased across assessment points (effect sizes of 0.66, 0.32, 0.46, 0.42, and 0.40 for t1–t5, respectively). Typically developing children's performance improved significantly (*p*s < 0.01) between t1 and t3/t4/t5, t2 and t4/t5, as well as t3/t4 and t5. Dyscalculic children showed significant improvements between all assessment points.

The 0–1000 number line condition (lower section of Figure 8) was not given at t1 as it was assumed to be too difficult for children at the beginning of Grade 2. Once again, we found significant main effects for assessment point, *F*_(1.78, 140.89)_ = 61.17, *p* < 0.01; η^2^ = 0.43 and arithmetic level as well as an interaction, *F*_(1, 81)_ = 30.94, *p* < 0.01; η^2^ = 0.28. Children with dyscalculia showed significantly larger deviances from the correct response than children with typical arithmetic development at all assessment points (*p*s < 0.01). The interaction resulted from the relatively high variability in t1 compared to later assessment points. Among typically developing children, significant improvements were observed between t2 and t5 as well as between all later assessment points. Dyscalculic children showed significant improvements between all four assessment points (all *p*s < 0.01).

In order to test earlier claims (Siegler and Booth, 2004) that children's mental number line progresses from a logarithmic to a linear representation over time, we calculated both regression lines separately for each child and each assessment point. No such developmental change could be observed for the typically developing children for whom a linear fit (*R*^2^s between 0.88 and 0.99) was found to describe children's performance better than a logarithmic fit (*R*^2^s between 0.69 and 0.79) at all assessment points and in both conditions (all *p*s < 0.01 in Wilcoxon signed-rank tests). For the dyscalculia group, a logarithmic fit (*R*^2^ = 0.71) seemed somewhat more adequate than a linear fit (*R*^2^ = 0.64, *p* < 0.01) at t1 where only the number line 0–100 was given, however, both fits were rather low. At all later assessment points, a linear fit (*R*^2^s between 0.89 and 0.99) described dyscalculic children's performance clearly better than a logarithmic fit (*R*^2^s between 0.70 and 0.76, all *p*s < 0.01).

Correlations between assessment points were moderate for both conditions among the first four assessment points (*r*s between 0.33 and 0.62, all *p*s < 0.005) and clearly higher between t4 and t5 (0.97 and 0.99)

## Discussion

In this study, typically developing and dyscalculic children's development of numerical processing was followed from the beginning of Grade 2 until the beginning of Grade 4. While most earlier studies investigating basic numerical processing in elementary school either compared different age groups cross-sectionally or covered relatively short periods of development, the present design allowed us to examine intra- as well as interindividual differences in numerical processing during a 2-year period which constitutes an important phase of arithmetic development. In these elementary school years, the foundations of arithmetic like place-value system, mental arithmetic, and written calculations are taught and practiced in school. Numerical processing has been demonstrated to be associated with these arithmetic skills (Halberda and Feigenson, 2008; de Smedt et al., 2009; Jordan et al., 2009; Geary, 2011), which is why it seemed particularly interesting to focus on this developmental period. Another crucial reason for the focus on these early school years was that this is when problems in arithmetic development become obvious and dyscalculia is diagnosed. For the present purpose, we selected groups of children who showed either age-adequate or persistently poor arithmetic performance during the study period. Marked and persistent problems in arithmetic in spite of adequate general cognitive abilities are the central diagnostic criterion of dyscalculia (e.g., World Health Organization, 2010). It is as yet unclear which subcomponents of numerical processing are central to arithmetic development, therefore we decided to apply a battery of tasks that have been used before to assess standard effects of symbolic as well as non-symbolic numerical processing.

### Indicators of numerical development during elementary school

A first finding was that all investigated effects of numerical processing were evident as early as Grade 2 for both typically developing children and children with persistent arithmetic problems. This is mostly consistent with earlier studies (e.g., Sekuler and Mierkiewicz, 1977; Girelli et al., 2000; Landerl and Kölle, 2009; Pixner et al., 2009; Reeve et al., 2012). It was, however, surprising for the physical comparison task. The size-congruity effect requires a certain amount of experience-based automaticity in numerical processing. It is influenced by certain task characteristics (most importantly the difference in physical size between the two presented digits, see Schwarz and Ischebeck, 2003) and earlier studies already indicated that there is a good deal of variability in when it appears. Importantly, in the current study, not only typically developing but also dyscalculic children showed sufficient automaticity in numerical processing to produce facilitation and interference effects even at the beginning of Grade 2. Together with the finding that there was no increase of these effects over time we confirmed Bugden and Ansari's (2011) claim that automatic processing of Arabic numerals is not directly related to arithmetic skills.

Generally, efficiency of numerical processing turned out to be a very good indicator of numerical development. Throughout the study period, we observed a systematic increase in speed of processing for numbers, which was larger than the general increase in processing speed that is characteristic for child development (Kail, 1991). Furthermore, the dyscalculia group showed persistent deficiencies in the speed of processing which were specific to numerical information and did not extend to non-numerical comparisons.

While efficiency of numerical processing improved consistently, many of the investigated within-task effects of numerical processing remained largely constant across time, for both typically developing and dyscalculic children. More specifically, while some earlier cross-sectional studies had suggested that the distance effect would decrease over time indicating an incremental specification of the cognitive representation of number (Holloway and Ansari, 2008; Landerl and Kölle, 2009), our longitudinal data showed no such decrease, neither for the symbolic nor for the non-symbolic comparison task. It might be argued that in relation to the decreasing intercepts, slopes that remain constant across time in fact indicate a relative increase of the investigated effect. In other words, when overall numerical processing becomes more efficient during development, it could be expected that within-task effects should decrease in accordance with intercepts, while our evidence suggests that slopes did not change much. However, even when within-task effects (slopes) were expressed as changes of IE-scores relative to overall efficiency of numerical processing (intercepts), no significant differences appeared across assessment points or arithmetic level, which confirms that the symbolic and non-symbolic distance effects did not undergo marked changes during the study period. The most likely explanation for this negative evidence is that within-task effects were relatively small while intra- as well as interindividual variability of task performance was relatively high. Correspondingly, stability was found to be low until middle of Grade 3.

### Stability of numerical processing

Although we found moderate to medium-range correlations across assessment points for intercepts, correlations for experimental effects were mostly non-significant. Only between the last two assessment points of our study period, i.e., middle of Grade 3 and beginning of Grade 4, robust correlations were evident for all measures. In summary, significant long-term stability could be observed for the overall efficiency of numerical processing. Yet, reasonable stability of within-task effects of numerical cognition was only achieved toward the end of primary school, but was found to be low in the early phases. Note that the 83 participants of the present study were specifically selected because their arithmetic competence showed a relatively steady development over time. It is likely that stability of numerical processing is even lower in the full sample of 139 children (including those who showed a variable arithmetic profile) and in the general population. Reeve et al. (2012) have recently reported reasonable stability for a random sample across a 6-year period for dot enumeration and symbolic number comparison. This analysis was also mostly based on speed of response and limited to ordinal correlations of group membership. Reeve et al.'s finding that 69% of the sample remained in the identified “slow,” “medium,” and “fast” groups implicates that almost one third of the participants exhibited considerable variability in their numerical processing development.

### Dyscalculia

A main research question of the current study was whether the numerical development of children with persisting arithmetic problems is mostly delayed or whether it would be possible to identify dyscalculia-specific anomalies in numerical cognition. As already mentioned, the dyscalculic children showed serious and pervasive deficiencies with respect to efficiency of numerical processing. Importantly, these problems were not limited to those tasks that required processing of symbolic representations of number, but were also evident for the magnitude comparison task. Thus, the current data do not provide support for Rousselle and Noël's (2007) proposal of specific problems to access numerical information from symbolic representations. However, note again that although the dyscalculia sample performed at a systematic lower level in the comparison paradigms, they showed symbolic as well as non-symbolic distance effects that were not significantly different from the typically developing children. Thus, we could not confirm Mussolin et al.'s (2010) finding of stronger symbolic and non-symbolic distance effects in dyscalculia. It is possible that problems to differentiate between two numbers or quantities are more prominent for smaller distances due to higher numerical similarity. This might explain as to why the Mussolin et al. study, which examined distances up to only four, found a reliable difference that we did not detect. Furthermore, their sample was somewhat older (10–11 years) and smaller and may have performed more homogeneously than the current sample. We can also not rule out that a ratio-based design of magnitude comparison might have revealed lower acuity of the approximate number system as it was reported before (see Piazza et al., 2010). Based on the findings of the current repeated assessment of the distance effect, we conclude that although dyscalculic children have marked problems to access their numerical cognition system efficiently, we did not find evidence for abnormal cognitive representations of numerosities in symbolic and non-symbolic comparison paradigms.

Anomalies were, however, evident in the dot enumeration paradigm where the dyscalculia group showed not only larger intercepts, but also persistently larger slopes in the subitizing range. For the higher numbers of the counting range, group differences in slopes were less marked. This evidence provides further support for earlier claims of a particular subitizing problem in dyscalculia (Moeller et al., 2011; Schleifer and Landerl, 2011; Reeve et al., 2012). Butterworth (2010) has argued that subitizing may reflect an inborn capacity to quantify over sets which provides the foundation for associating numbers with distinct numerosities. Such an early deficit may well induce problems in mapping between numbers and quantities and in the long run a general inefficiency in numerical processing as it was observed in the current data set. Over time, it would also induce a general imprecision of numerical representations. This is exactly what we found in the number line task: Over the whole study period, dyscalculic children showed larger deviances from the precise location of a number on a number line than children with typical development of arithmetic skills. The number line task is particularly important in the current design as it was the only untimed task in our numerical processing battery. The persistent deficit in the dyscalculia sample shows that their numerical processing problems are not limited to processing speed. Interestingly, earlier claims of a developmental trajectory from an overrepresentation of small numbers in the mental number line inducing a logarithmic function to a linear representation (e.g., Booth and Siegler, 2006), did not find support in the current data set (see also Landerl et al., 2009).

An important aspect that has not yet been thoroughly investigated in dyscalculia is the acquisition of the place-value system of the Arabic notational system. Our findings on processing of two-digit numbers add to current evidence on young children's difficulties to integrate ten and unit numbers (Nuerk et al., 2004; Pixner et al., 2009; Mann et al., 2011, 2012). In accordance with Pixner et al. (2009) we found particularly poor performance at the beginning of Grade 2, but rapid improvement in the competence to process two-digit numbers for the typically developing group. Dyscalculic children's problems were clearly more pronounced and persistent throughout the study period. At t1 they actually chose the incorrect, smaller number in about half of the trials. One might assume that they attempted to select the larger number based on the unit number which would induce systematically wrong choices in the incompatible condition. However, the fact that they chose the larger unit digit in about half of the items in the incompatible condition (and therefore responded correctly) and even in about 15% of the compatible items (and therefore responded incorrectly) speaks against such a strategy and rather suggests that they were guessing. Note that in the German test-language, tens and units are inversed in numberwords (21 is “one and twenty”) which has been demonstrated to amplify children's problems to acquire the place-value system (Pixner et al., 2011).

### Implications for future research and dyscalculia diagnosis

In summary, the current longitudinal data set shows that efficiency of numerical processing is an important indicator of numerical skills: Despite considerable improvements during the elementary school years it remains persistently deficient in children with dyscalculia. While significant stability was found for speed, many of the investigated within-task effects were of low stability and not subject to developmental processes. Because of the low stability of these effects across time, they do not seem appropriate for diagnostic tests of dyscalculia. The most obvious criterion to identify children who struggle with their numerical processing system is the efficiency of numerical processing. It will be important to devise more computerized tests enabling accurate measurement of response times as this is the main indicator of efficiency of numerical processing in simpler tasks like dot enumeration of number comparison.

While the finding of a generally lower efficiency of numerical processing suggests a delayed rather than a deviant numerical development in dyscalculia, the current study also helped to identify parameters that go beyond the developmental delay perspective: The dyscalculia sample showed persistently larger slopes in the subitizing range of dot enumeration, inaccurate numerical estimation in the number line task and serious problems to integrate the component numerals in multi-digit numbers. Subitizing seems to have a strong biological basis (Vetter et al., 2011) and may be a very early indicator of a faulty numerical processing system, while both, the number line task and processing of multi-digit numbers, develop as a consequence of education and experience. While the focus of the current study was the development of numerical processing in elementary school children who already experience persistent problems in arithmetic, future studies should concentrate on earlier phases of development in order to identify the developmental trajectories of the relevant parameters even before the problems in arithmetic arise.

### Conflict of interest statement

The author declares that the research was conducted in the absence of any commercial or financial relationships that could be construed as a potential conflict of interest.

## Footnotes

^1^Ten children of the dyscalculia group performed more than 1.5 *SD*s below norm on all three assessment points, four children's performance was more than 1.5 *SD*s below norm on two assessments of arithmetic skills and 15 children performed more than 1.5 *SD*s below norm at least once during the study period. Only seven children of the dyscalculia group showed persistent but somewhat milder arithmetic deficits with performance lower than 1 *SD* below norm at the end of first grade and lower than 0.5 *SD*s below norm at the later assessment points.

^2^IE-scores for eight dots were excluded because previous studies (e.g., Trick and Pylyshyn, 1993) demonstrated end effects for the enumeration of the largest numerosity of a set. IE-scores for four dots were also excluded, as earlier studies (Schleifer and Landerl, 2011; Reeve et al., 2012) suggested that in this intermediate range, some children might still be able to subitize, while others would already resort to their counting skills.

^3^Due to unrealistic scores at t3, one dyscalculic child had to be excluded from analysis of the digit comparison task.

^4^As the task design was not based on ratios between the two displays, calculation of Weber fractions was not possible.
